# A Database of Force-Field Parameters, Dynamics, and Properties of Antimicrobial Compounds

**DOI:** 10.3390/molecules200813997

**Published:** 2015-08-03

**Authors:** Giuliano Malloci, Attilio Vittorio Vargiu, Giovanni Serra, Andrea Bosin, Paolo Ruggerone, Matteo Ceccarelli

**Affiliations:** Dipartimento di Fisica, Università degli studi di Cagliari, Cittadella Universitaria, I-09042 Monserrato (Cagliari), Italy; E-Mails: giuliano.malloci@dsf.unica.it (G.M.); gserra@dsf.unica.it (G.S.); andrea.bosin@dsf.unica.it (A.B.); paolo.ruggerone@dsf.unica.it (P.R.); matteo.ceccarelli@dsf.unica.it (M.C.)

**Keywords:** antimicrobial compounds, molecular databases, all-atom force fields, molecular dynamics simulations

## Abstract

We present an on-line database of all-atom force-field parameters and molecular properties of compounds with antimicrobial activity (mostly antibiotics and some beta-lactamase inhibitors). For each compound, we provide the General Amber Force Field parameters for the major species at physiological pH, together with an analysis of properties of interest as extracted from μs-long molecular dynamics simulations in explicit water solution. The properties include number and population of structural clusters, molecular flexibility, hydrophobic and hydrophilic molecular surfaces, the statistics of intra- and inter-molecular H-bonds, as well as structural and dynamical properties of solvent molecules within first and second solvation shells. In addition, the database contains several key molecular parameters, such as energy of the frontier molecular orbitals, vibrational properties, rotational constants, atomic partial charges and electric dipole moment, computed by Density Functional Theory. The present database (to our knowledge the first extensive one including dynamical properties) is part of a wider project aiming to build-up a database containing structural, physico-chemical and dynamical properties of medicinal compounds using different force-field parameters with increasing level of complexity and reliability. The database is freely accessible at http://www.dsf.unica.it/translocation/db/.

## 1. Introduction

The study of dynamical interactions between drugs and their biological targets (such as nucleic acids and proteins) is of paramount importance in medicinal chemistry and related fields (see e.g., [1,2]). Among the many tools, numerical computations including molecular dynamics (MD) simulations have gained an ever increasing role in addressing key structural, dynamical, thermodynamic and kinetic features at a molecular level of detail [3,4,5,6,7,8,9,10,11]. Furthermore, thanks also to the steep increase in computational power (e.g., the recent porting on GPUs allowed the all-atom simulations of porins embedded and solvated in membrane beyond the s timescale [12]), MD simulations are no longer a mere complement to experiments, but truly inspire new research lines and experimental work [5,6,13,14,15,16].

Dynamical interactions can be described in MD simulations by means of different models (see e.g., [17]): from full-quantum descriptions further approximations lead to mixed classical-quantum (QM/MM), all-atom, mixed molecular mechanics/coarse-grained (MM/CG) and coarse-grained (CG) models to represent “atomic” interactions.

In particular, MD simulations based on all-atom empirical “force-fields”, nowadays routinely performed in the microsecond timescale, have been proven to yield structural, dynamical, thermodynamical, and kinetic information with good accuracy [5,14,15]. Irrespective of the physical model used to describe interatomic forces, the key ingredient of empirical MD simulations are the parameters entering terms of the force-field, which are usually fitted to reference experimental or theoretical data [18]. The use of the latter scheme, facilitated by the introduction of the Amber 95 force field [19], allowed to improve considerably the range of application/accuracy of empirical simulations. The possibility to perform high quality *ab-initio* calculations has indeed allowed the fitting to any property, including those not or partly accessible through experiments. An example in this direction was the use of DFT to calculate all vibrational frequencies and related eigenvectors of a chromophore [20], that once reproduced by the empirical parameters has opened the way to investigate more precisely the elecronic-vibrational coupling in photosynthesis [21]. The force-fields widely used today in biomolecular simulations have reached a very good level of description of structural and dynamical properties of common macromolecules such as proteins [22], nucleic acids [23], and membranes [24], although corrections and improvements have been recently proposed [25]. However, the parametrization of generic molecules (drugs, dyes, *etc*.) remains often a non-trivial task [26], despite the efforts in developing (semi-)automatic parametrization tools. Among the widely used public databases and tools are the R.E.DD.B. [27,28] and the AMBER parameter [29] databases. Obtaining reliable force-fields for general molecules requires the combination of several tools and expertise, from chemical characterization [30] to classical [31] and/or quantum calculations [32] at different levels, as well as chemical, physical, and biological intuition.

Recently, as part of the activity of the TRANSLOCATION consortium within the Innovative Medicines Initiative antimicrobial resistance programme, New Drugs for Bad Bugs [33], we have undertaken a long-term project with the goal of building a large database of antimicrobial compounds (and medicinal compounds in general) containing, for each molecule, all-atom parameters compatible with different existing biological force fields, as well as microsecond-long dynamics and physico-chemical descriptors in different physiological conditions. Thus, using several established computational tools we have started a systematic investigation on antimicrobial compounds of different classes. Besides the specific application to the study of bacterial resistance mediated by the membrane barrier [34,35], our database contains molecular information possibly useful in different contexts such as, for example, receptor-ligand molecular docking and indirect assessment of role of flexibility in the interpretation of experimental results (see Section 4).

This work constitutes the first step of the aforementioned wider project: we report a homogenous database of the computed molecular properties for a sample of 40 different antimicrobial compounds ranging in size from ∼20 to ∼80 atoms (see Table A1 in the Appendix). For each compound, we provide the General Amber Force Field parameters [36] for the major species at physiological pH, together with an analysis of properties of general interest, including number and population of relevant structural clusters, molecular flexibility, hydrophobic and hydrophilic molecular surfaces, as well as the statistics of intra- and inter-molecular H-bonds, hydration shells structure and dynamics, *etc*., as extracted from μs-long molecular dynamics simulations in explicit water solution. In addition, the database includes several key molecular parameters, such as energy of the frontier molecular orbitals, normal modes of vibration, rotational constants, atomic partial charges and electric dipole moment, computed by Density Functional Theory. This database is freely accessible on-line at the address http://www.dsf.unica.it/translocation/db/ and is suitable for further inclusion of new data.

## 2. Computational Methods

For each compound we obtained the 3D structure data file (SDF format) from the Pubchem database [37]. We then used the ChemAxon’s Marvin suite of programs [30] to calculate the dominant tautomer distribution and thus find the protonation/charge state most populated at physiologicalpH = 7.4. We used the same package to obtain other general properties of interest, such as the net charge dependence on pH, the isoelectric point pI, the Van der Waals volume, the number of rotatable bonds, and the number of H-bond donors/acceptors.

### 2.1. QM Calculations

The structure of the major species determined in the above step has been subsequently used as an input to quantum-chemical calculations at the Density Functional Theory level [38]. For this part of the work we used the Gaussian09 package [32]. In particular, we employed the widely used hybrid B3LYP functional, a combination of exact (Hartree-Fock) exchange with local and gradient-corrected exchange and correlation terms [39,40], in conjunction with the split-valence6-31G${}^{★★}$ Gaussian basis-set [41]. The combinationB3LYP/6-31G${}^{★★}$ represents a good compromise between accuracy and computational cost [42,43]. In all cases considered we disabled the use of molecular symmetry (Symmetry=None), adopted very restrictive convergence criteria for both self-consistent-field iterations (10${}^{-8}$ Ha, SCF(Conver=8)) and geometry optimizations (Opt(VeryTight)), and used a pruned (99,590) grid (Int=UltraFine) for numerical integration. For each compound we optimized the ground-state structure employing the Polarizable Continuum Model [44] as to mimic the effect of water solvent (SCRF=(PCM, Solvent=Water)) particularly to avoid formation of strong intra-molecular H-bonds. To confirm the geometry obtained to be a global minimum on the potential energy surface we performed full vibrational analyses obtaining real frequencies in all cases. We processed the output of Gaussian09 with GaussSum [45] and Gabedit [46] to extract orbital data, and vibrational spectra, respectively.

On the optimized geometry we then performedB3LYP/6-31G${}^{★★}$ single-point energy calculations in vacuum to generate the atomic partial charges fitting the molecular electrostatic potential. Under the constraint of reproducing the overall electric dipole moment of the molecule, we used both Merz-Kollman [47] (Pop=MK) and CHELPG [48] (Pop=CHelpG) schemes to construct a grid of points around the molecule. Atomic partial charges were then generated through the two-step restrained electrostatic potential (RESP) method [49] implemented in the Antechamber package [50]. Following the same procedure we additionally extracted the standardHF/6-31G* point charges, which are fully compatible with the charge derivation protocol within AMBER [49]. The derivation of consistent charges is hardly a straightforward task for which specifically devoted tools and databases have been developed, such as the R.E.DD.B. database to give an example [27,28]. Finally, for theB3LYP/6-31G${}^{★★}$ optimized geometry, we computed logP values using the XLOGP3 program [51], and polar/non-polar molecular surfaces with the PLATINUM web interface [52].

### 2.2. MD Simulations

We performed all-atom molecular dynamics simulations in the presence of explicit water solution (0.1 M KCl) using the Amber14 package [31]. Model systems were prepared with the program *tleap* of AmberTools14 [31] adopting the TIP3P model of water [53] and the monovalent ion parameters appropriate for this choice [54]. For the antibiotics we used the General AMBER Force Field (GAFF) parameters [36]. For all systems under investigation we used the following procedure. First, geometry optimization was conducted with a two-step protocol: (i) 10,000 cycles (1000 of steepest descent plus 9000 of conjugate gradient) with harmonic restraint k = 10 kcal · mol${}^{-1}$· Å${}^{-2}$ on each heavy atom of the solute and (ii) 10,000 conjugate gradient cycles without restraints. Next, an heating to 400 K followed by a cooling to 310 K were accomplished via NVT MD runs for 2 ns and 10 ns, respectively. As a last step preceding the productive dynamics, NPT MD was conducted for 1 ns to relax the simulation box. Finally, 1 μs-long MD simulations were performed under the NPT ensemble. Pressure and temperature were regulated at 1 Atm and 310 K using the isotropic Berendsen barostat [55] and the Langevin thermostat [56], respectively. Electrostatic interactions were evaluated using the particle mesh Ewald scheme with a cutoff of 9.0 Å for the short-range evaluation in direct space. The same cutoff was used for Lennard-Jones interactions (with a continuum model correction for energy and pressure) [31].

### 2.3. Post-Processing of the MD Trajectories

**Figure 1:**
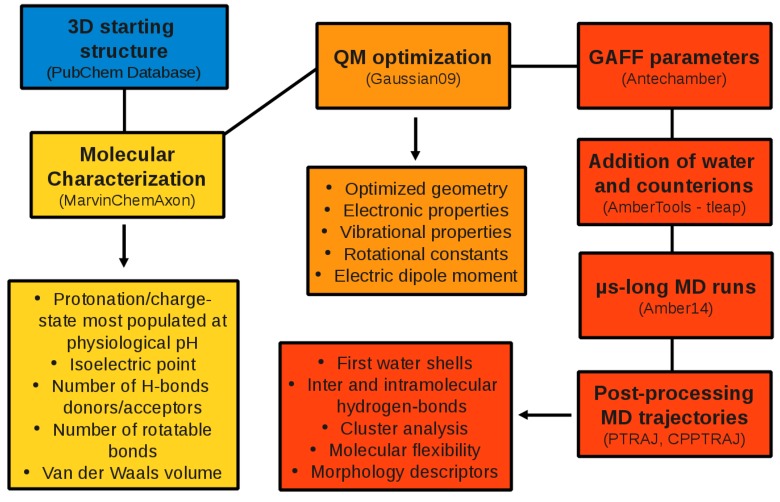
Computational protocol adopted (See Section 2 for a description).

From the all-atom MD simulations we obtained structural and dynamical features of the compounds investigated by means of the PTRAJ and CPPTRAJ programs [57]. In detail, we extracted first and second water shells using a lower (upper) cutoff of 3.4 (5.0) Å. For the analysis of intra- and inter-molecular H-bonds we adopted angle and distance cutoffs of 135${}^{\circ }$ (donor-hydrogen-acceptor angle) and 3.5 Å (donor-acceptor), respectively [58,59,60,61,62]. The number and population of structural clusters were determined using a hierarchical agglomerative algorithm [63]. To evaluate atomic root mean square fluctuations we used the utility g_rmsf of GROMACS [64]. During the MD runs we also monitored three morphology descriptors related to the gyration tensor, *i.e*., asphericity, acylindricity, and kappa2, as implemented in the PLUMED plugin [65]. Asphericity and acylindricity give a measure of the deviation of the mass distribution from spherical and cylindrical symmetry, respectively; the relative shape anisotropy kappa2 is limited between 0 and 1 and reflects both symmetry and dimensionality [66]. The dynamical evolution of the minimal projection area has been determined with the combined use of Open Babel [67] and ChemAxon’s Calculator Plugin [30]. Molecular graphics have been generated by using the PyMOL [68] and VMD [69] packages. The overall computational protocol adopted is schematically depicted in Figure 1.

## 3. Results

### 3.1. General Structure of the Database

The total of 40 species presently included in the database, ranging in size from clavulanic acid (22 atoms, molecular weight = 198.1528 Da) to tigecycline (82 atoms, molecular weight = 586.6566 Da), covers eight different classes of antibiotics and related compounds, namely: carbapenems, cephalosporins, monocyclic beta-lactams, oxazolidinones, penicillines, quinolones, tetracyclines, and beta-lactamase inhibitors. Antimicrobial compounds of these classes are the most widely used against infections caused by Gram-negative bacteria, such as *Escherichia coli*, *Pseudomonas aeruginosa*, *Salmonella aenterica* and *Klebsiella pneumoniae*. The complete set of compounds included in the current version of the database, together with some of their general properties are listed in Table A1 of the Appendix. Most of the parameters we provide play a key role in determining the translocation of small molecules, such as antibacterials, through bacterial porins [70,71,72], as well as their extrusion by efflux pumps [73,74,75], which are among the key topics of the New Drugs for Bad Bugs programme [33].

**Figure 2:**
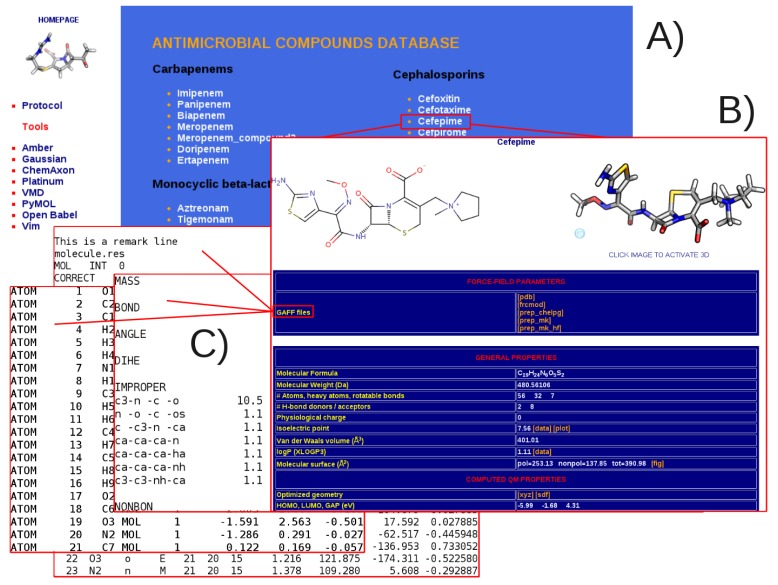
Snapshots of the main page of the database (**A**); an individual page (**B**); and the General Amber Force Field parameters files downloadable for each compound (**C**).

The main-page http://www.dsf.unica.it/translocation/db/ contains the full list of compounds ordered, within each class, by increasing molecular weight (see Figure 2A). As shown in the left side of the picture, a direct link to the official web-page of all of the computer packages and tools employed is also given. A separate page can then be accessed for each given molecule (Figure 2B). The latter includes a 2D representation of the selected molecule as well as the corresponding QM-optimized 3D structure that can be interactively manipulated by activating a JSmol script [76]. The General AMBER Force Field (GAFF) parameters files (.prep and .frcmod formats [36]) generated with the AmberTools package [31] can be downloaded within each compound’s page (Figure 2C). We provide three sets of atomic point-charges computed in vacuum: the standard HF/6-31G(d) (molecule_mk_hf.prep) and the B3LYP/6-31G(d,p) charges fitting the molecular electrostatic potential using the CHELPG (molecule_chelpg.prep) and Merz-Kollman (molecule_mk.prep) schemes (see Section 2).

**Figure 3:**
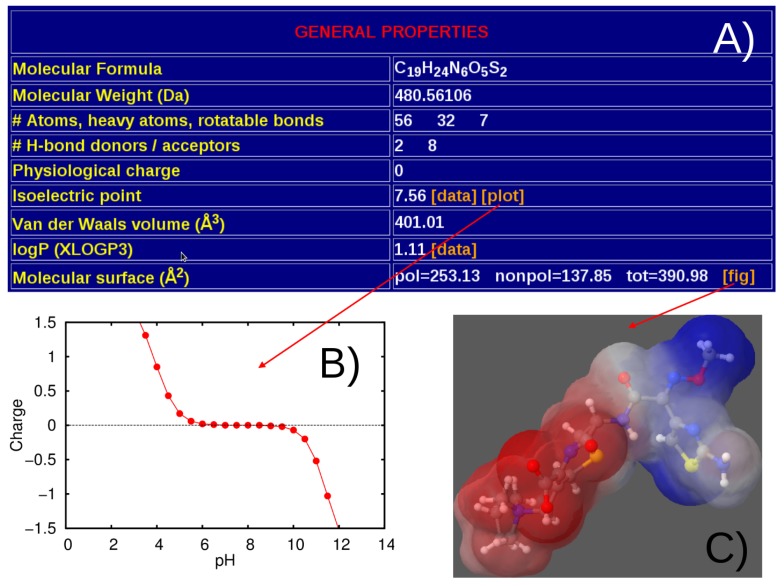
Snapshots of the general properties available for each compound (**A**); the molecular net charge dependence on pH computed with MarvinSketch [30] (**B**); and the polar/non-polar (red-white-blue color scale) molecular surfaces evaluated with the PLATINUM server [52] (**C**).

**Figure 4:**
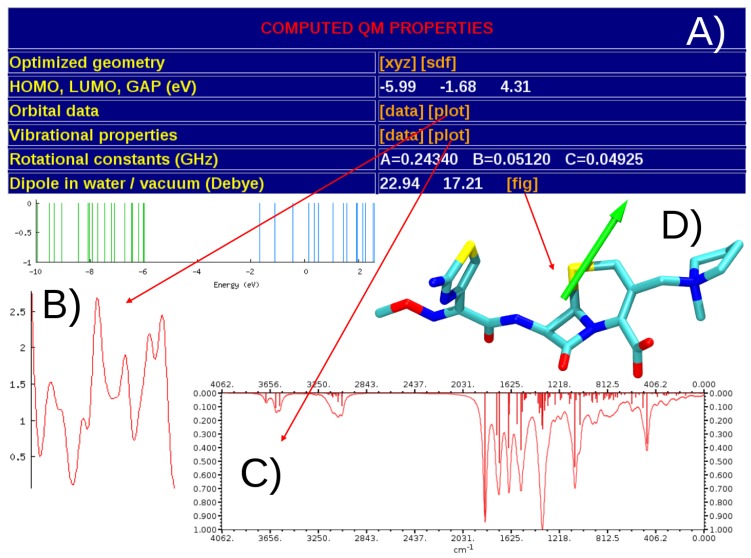
Snapshots of the list of molecular properties exctracted from QM calculations (**A**); the electronic (**B**); and vibrational spectra (**C**); and the visual representation of the electric dipole moment (**D**).

A separate table reports general properties as well as molecular descriptors extracted from both quantum-mechanics and molecular-dynamics simulations. As shown in Figure 3, among general properties we report molecular formula, molecular weight, number of atoms and rotatable bonds, number of H-bond donors/acceptors, physiological charge, Van der Waals volume, isoelectric point pI and pH-dependent net charge of the molecule. For the quantum-optimized structure we provide also logP values [51], and polar/non-polar molecular surfaces [52]. In the quantum-mechanical section (Figure 4) the ground-state optimized geometry is available in both plain .xyz and MDL .sdf formats. For the above molecular structure we report: energy of the highest-occupied molecular orbital (HOMO) and lowest-unoccupied molecular orbital (LUMO), HOMO-LUMO gap, rotational constants, and electric dipole moment computed in vacuum and implicit water. The whole set of orbital data and the corresponding plots (density of electronic states, virtual and occupied levels) extracted with GaussSum [45] (Figure 4B), as well as the list of harmonic vibrational frequencies and integrated absorption coefficients with the corresponding IR absorption spectrum obtained with gabedit [46] are also provided (Figure 4C). A graphical representation of the spatial orientation of the electric dipole is additionally given (Figure 4D).

**Figure 5:**
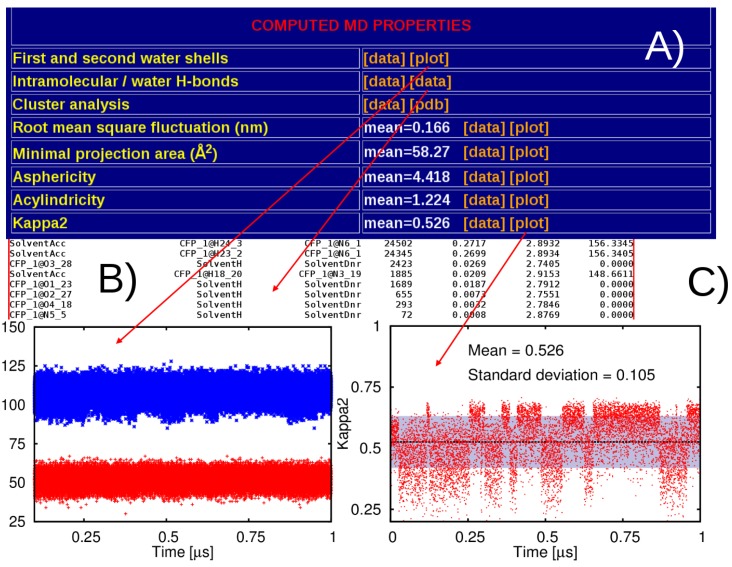
Snapshots of the some of the molecular properties exctracted from MD trajectories (**A**); as an example we report first and second water shells and statistics of solute-solvent H-bonds (**B**), and the dynamical behaviour of the relative shape anisotropy (**C**).

Figure 5 offers a visual representation of some of the molecular descriptors extracted from the MD simulations. The complete set includes: number of solvent molecules within first and second solvation shells, the statistics of intra- and inter-molecular H-bonds, number and population of structural clusters, molecular flexibility expressed in terms of root mean square fluctuations, as well as dynamical behavior of asphericity, acylindricity, and kappa2.

## 4. Discussion

Among the antimicrobial compounds included in this release of the database, those for which *all* of the above data are simultaneously available from experiments is relatively small. Thanks to the ever increasing computational power available, the use of modeling tools represents the best alternative to obtain homogenously-derived physico-chemical descriptor of molecules, and can be furthermore useful as a guide for future experimental work. There are many Internet resources reporting relevant data for a large number of compounds of medicinal interest (PubChem, ChemSpider, DrugBank, Chemicalize, *etc*.). However, to the best of our knowledge, the present database is the first extensive one reporting structural, physico-chemical, and especially *dynamical* properties obtained combining different computational tools (in particular μs-long long MD simulations). There are basically three different levels of active use of our data. First of all the obvious stand-alone use of the tabulated static and dynamical properties of selected compounds. Second, the availability of the (at the moment only) GAFF parameters for the major microspecies at physiological pH, makes possible to straightforwardly perform MD simulations with ready-to-use input files. Finally, the knowledge of the dynamical behavior of a large number of molecular descriptors extracted from μs-long MD runs allows performing statistical analyses that go beyond the availability of one static value. This piece of information can find a plethora of possible applications, for example in the field of molecular docking, just to mention one. Most of the available docking programs take into account ligand flexibility only in terms of rotatable bonds of *one* given input structure (see e.g., rDock [77] and AutoDock Vina [78]) or in terms of small deformations around an equilibrium conformation, derived e.g., by applying normal modes or related analyses [79]. However, as largely demonstrated particularly in the case of protein-protein interactions [80,81,82,83,84,85,86,87], the availability of different conformations sampled by the ligand during MD simulations can be crucial also (perhaps *mostly*) in protein-ligand docking [73,86,88,89,90], considerably improving the protocol and making possible to find otherwise unaccessible binding conformations. This is particularly true in the context of conformational selection theory [7], where conformations having low probability in the absence of receptor are populated by the onset of ligand-protein interactions.

The knowledge of the values distribution for specific molecular descriptors can be also useful in the study of structure-dynamics-activity relationships by means of specific molecular descriptors. For instance, the minimal projection area (MPA) has been recently used [91] in the context of bacterial resistance mediated by Resistance-Nodulation Division (RND) multidrug efflux transporters [92,93,94,95,96,97], to identify a correlation between reduced efflux activity by mutants of the transporter AcrB and the size of substrates. Table 1 reports the MPAs computed for the smallest and largest compound within each of the eight classes considered in the present work. The table reports the *dynamical* values extracted from the MD trajectories and compares them with the *static* ones publicly available from Chemicalize [98]. As shown in Table 1 the value reported in Chemicalize fall in the Mean ± Stdev range in almost all cases considered; a relevant exception is represented by ertapenem for which Chemicalize predicts the corresponding MPA to be larger than about 26% with respect to our mean value. Taking into account the minimum and maximum values and the corresponding range of variability, the distribution of the computed MPAs displays large deviations (from ∼10% up to ∼40%) around the mean value. Thus, our data can be used to take into account the dynamical nature of interacting partners when studying ligand transport processes, as well as any interaction dynamics between antibiotics and their targets.

**Table 1:** Comparison between minimal projection areas taken from Chemicalize [98] and the average values and range of variability extracted from MD runs for selected compounds.

Compound	Minimal Projection Area (Å${}^{2}$)		
	Chemicalize	Mean ± Stdev	Min, Max (Delta)
Carbapenems			
Imipenem	n.a.	44.7 ± 3.2	35.4, 56.6 (21.2)
Ertapenem	72.6	57.4 ± 6.3	43.3, 82.5 (39.2)
Cephalosporins			
Cefoxitin	59.8	57.8 ± 5.2	41.9, 70.1 (28.2)
Ceftazidime	66.0	68.3 ± 5.7	52.8, 88.1 (35.3)
Monocyclic beta-lactams			
Aztreonam	59.6	58.4 ± 2.7	48.3, 69.3 (21.0)
BAL30072	n.a.	64.7 ± 4.7	50.2, 81.4 (31.2)
Oxazolidinones			
Linezolid	47.6	43.4 ± 4.0	32.5, 53.5 (21.0)
Sutezolid	n.a.	44.0 ± 3.9	32.6, 56.8 (24.2)
Penicillines			
Aminopenicillanic acid	39.9	38.1 ± 0.7	35.0, 41.5 (6.5)
Piperacillin	86.6	77.5 ± 3.6	56.1, 90.0 (33.9)
Quinolones			
Nalidixic acid	34.3	36.5 ± 1.4	32.6, 42.7 (10.1)
Fleroxacin	46.6	46.1 ± 1.9	39.8, 55.5 (15.7)
Tetracyclines			
Minocycline	66.2	67.5 ± 1.4	61.4, 72.9 (11.5)
Tigecycline	76.2	76.6 ± 3.5	65.5, 86.7 (21.2)
Beta-lactamase inhibitors			
Clavulanic acid	38.6	37.5 ± 1.1	32.1, 40.4 (8.3)
Tazobactam	47.0	44.2 ± 3.0	39.3, 52.5 (13.2)

## 5. Perspectives

In the near future we plan to extend the amount of information present in the database. For instance, in order to guarantee reproducibility, we plan to make available the input and output files of our simulations. Furthermore, the database will be extended towards two main directions. From one side we will include more compounds, covering additional antimicrobial classes. At the same time, for each compound, we will setup parameters at different pHs including at least two more cases (low and high-pH) in addition to the physiological pH already considered. We plan to include in the individual page of each compound the relevant literature reporting on molecular properties from both experimental and computational studies. From the technical point of view we plan to improve the key parameters reported, in particular dihedral angles, by comparison with quantum-mechanical dynamics in presence of implicit and explicit solvent molecules, using biased molecular dynamics techniques [99,100,101,102]. Future refinements of the database will include the possibility to use a better water model to perform classical MD simulations. Finally, future directions include also performing MD simulations in solvents different than water (e.g., non polar solvents and “organic broths”) in order to further improve conformational sampling while (possibly) mimicking interactions with functional groups in biomolecules such as proteins, nucleic acids, and membranes.

## Appendix A

**Table A1:** List of antimicrobial compounds included in the current version of the database.

Compound		Molecular	Number	Molecular	Physiological
		Formula	of Atoms	Weight	Charge
Carbapenems					
Imipenem		C_12_H_17_N_3_O_4_S	37	299.3461	0
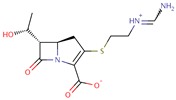
Panipenem		C_15_H_21_N_3_O_4_S	44	339.4099	0
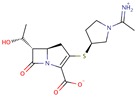
Biapenem		C_15_H_18_N_4_O_4_S	42	350.3928	0
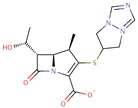
Meropenem		C_17_H_25_N_3_O_5_S	51	383.4625	0
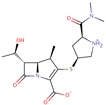
Meropenem 2		C_17_H_25_N_3_O_5_S	51	383.4625	0
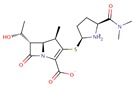
Doripenem		C_15_H_24_N_4_O_6_S	51	420.5043	0
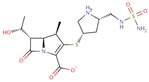
Ertapenem		C_22_H_24_N_3_O_7_S	57	474.5069	–1
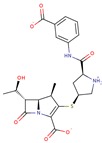
Cephalosporins					
Cefoxitin		C_16_H_16_N_3_O_7_S_2_	44	426.4441	–1
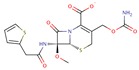
Cefotaxime		C_16_H_16_N_5_O_7_S_2_	46	454.4575	–1
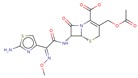
Cefepime		C_19_H_24_N_6_O_5_S_2_	56	480.5611	0
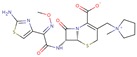
Cefpirome		C_22_H_22_N_6_O_5_S_2_	57	515.5773	0
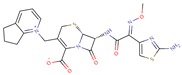
Ceftobiprole		C_20_H_22_N_8_O_6_S_2_	58	534.5687	0
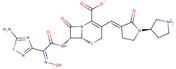
Ceftazidime		C_22_H_21_N_6_O_7_S_2_	58	545.5681	–1
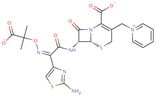
Monocyclic beta-lactams					
Aztreonam		C_13_H_15_N_5_O_8_S_2_	43	433.41690	–2
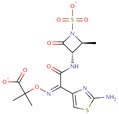
Tigemonam		C_12_H_13_N_5_O_9_S_2_	41	435.3897	–2
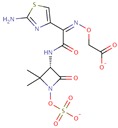
Carumonam		C_12_H_12_N_6_O_10_S_2_	42	464.3879	–2
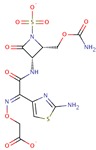
BAL19764		C_14_H_13_N_6_O_9_S_2_	44	473.4178	–1
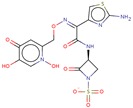
Nocardicin		C_23_H_22_N_4_O_9_	58	498.4422	–2
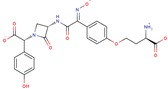
BAL300072		C_16_H_17_N_6_O_10_S_2_	51	517.47038	–1
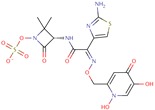
Oxazolidinones					
Linezolid		C_16_H_20_FN_3_O_4_	44	337.3461	0
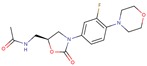
Sutezolid		C_16_H_20_FN_3_O_3_S	44	353.41170	0
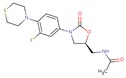
Penicillines					
Aminipenicillanic acid		C_8_H_11_N_2_O_3_S	25	215.2495	–1
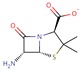
Benzylpenicillin		C_16_H_17_N_2_O_4_S	40	333.3822	–1
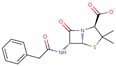
Ampicillin		C_16_H_19_N_3_O_4_S	43	349.4048	0
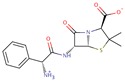
Carbenicillin		C_17_H_16_N_2_O_6_S	42	376.3837	–2
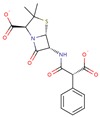
Ticarcillin		C_15_H14N_2_O_6_S_2_	39	382.4115	–2
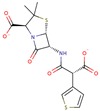
Oxacillin		C_19_H_18_N_3_O_5_S	46	400.4283	–1
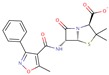
Piperacillin		C_23_H_26_N_5_O_7_S	62	516.5468	–1
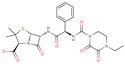
Quinolones					
Nalidixic acid		C_12_H_11_N_2_O_3_	28	231.2273	–1
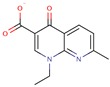
Norfloxacin		C_16_H_18_FN_3_O_3_	41	319.3308	0
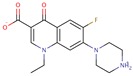
Ciprofloxacin		C_17_H_18_FN_3_N_3_O_3_	42	331.3415	0
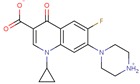
Enrofloxacin		C_19_H_21_FN_3_N_3_O_3_	47	358.3867	–1
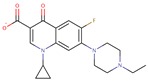
Levofloxacin		C_18_H_19_FN_3_N_3_O_4_	45	360.3596	–1
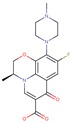
Fleroxacin		C_17_H_17_F_3_N_3_O_3_	43	368.3304	–1
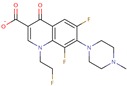
Tetracyclines					
Minocycline		C_23_H_27_N_3_O_7_	60	457.4764	0
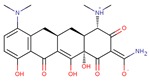
Tigecycline		C_29_H_40_N_5_O_8_	82	586.6566	1
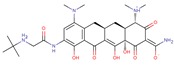
Beta-lactamase inhibitors					
Clavulanic acid		C_8_H_8_NO_5_	22	198.1528	–1
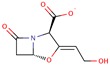
Sulbactam		C_8_H_10_NO_5_S	25	232.2337	–1
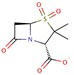
Avibactam		C_7_H_10_N_3_O_6_S	27	264.2358	–1
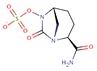
Tazobactam		C_10_H_11_N_4_O_5_S	31	299.2831	–1
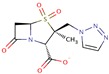

**Table A2:** Root-mean-square displacement between the B3LYP/6-31G${}^{★★}$ optimized geometry and the minimum-energy structure obtained with the GAFF parameters of the database.

Class	Compound	RMSD (Å)
Carbapenems	Imipenem	1.59
	Panipenem	1.70
	Biapenem	1.69
	Meropenem	1.66
	Meropenem-2	1.65
	Doripenem	1.66
	Ertapenem	1.71
Cephalosporins	Cefoxitin	1.63
	Cefotaxime	1.63
	Cefepime	1.62
	Ceftobiprole	1.64
	Ceftazidime	1.60
Monocyclic beta-lactams	Aztreonam	1.60
	Tigemonam	1.69
	Carumonam	1.64
	BAL19764	1.65
	Nocardicin	1.66
	BAL30072	1.76
Oxazolidinones	Linezolid	1.68
	Sutezolid	1.67
Penicillines	Aminopenicillanic Acid	1.73
	Benzylpenicillin	1.64
	Ampicillin	1.65
	Carbenicillin	1.68
	Ticarcillin	1.68
	Oxacillin	1.64
	Piperacillin	1.74
Quinolones	Nalidixic Acid	1.62
	Norfloxacin	1.58
	Ciprofloxacin	1.58
	Enrofloxacin	1.58
	Levofloxacin	1.56
	Fleroxacin	1.59
Tetracyclines	Minocycline	1.62
	Tigecycline	1.65
Beta-lactamase inhibitors	Clavulanic Acid	1.66
	Sulbactam	1.63
	Avibactam	1.62
	Tazobactam	1.66
